# Circadian disruption does not alter tumorigenesis in a mouse model of lymphoma

**DOI:** 10.12688/f1000research.125272.1

**Published:** 2023-01-12

**Authors:** Rebecca M Mello, Marie Pariollaud, Katja A Lamia

**Affiliations:** 1 Molecular Medicine, Scripps Research Institute, La Jolla, CA, 92037, USA

**Keywords:** c-MYC, circadian rhythm, lymphoma, circadian disruption, chronic jetlag, CRY2

## Abstract

**Background:** Disruption of natural diurnal light cycles, such as that experienced by shift workers, is linked to enhanced cancer incidence. Several mouse models of cancer have been shown to develop more severe disease when exposed to irregular light/dark cycles, further supporting the connection between circadian disruption and increased cancer risk. Cryptochrome 2 (CRY2), a repressive component of the molecular circadian clock, facilitates the turnover of the oncoprotein c-MYC, one mechanism that may link the molecular clock to tumorigenesis. In Eμ-MYC mice, which express transgenic *c-MYC *in B cells and develop aggressive lymphomas and leukemia, global *Cry2 *deletion reduces overall survival and enhances tumor formation. Moreover, lighting conditions that mimic the disruption experienced by shift workers dampens *Cry2 *transcripts in peripheral tissues of C57BL/6J mice. Thus, we hypothesized that exposure to disruptive lighting conditions would enhance tumor burden in Eμ-MYC mice.

**Methods:** We housed Eμ-MYC mice in light-tight boxes set to either the control (continuous cycles of 12-hours of the light followed by 12-hours of dark, LD12:12) or chronic jetlag (eight-hour light phase advances every two to three days, CJL) lighting conditions and assessed the impact of disrupted light cycles on overall survival and tumor formation in Eμ-MYC mice.

**Results:** Environmental disruption of circadian rhythms did not alter tumor location, tumor growth, or overall survival in female or male Eμ-MYC mice.

**Conclusions:** Our findings support emerging evidence that suggests the impact of circadian disruption on tumorigenesis is dependent on the origin of malignancies.

## Introduction

Circadian rhythms describe the diurnal oscillation of behavior and physiology in anticipation of environmental fluctuations. In mammals, lighting cues are transmitted from the retina, via the retinohypothalamic tract, to the central clock located in the suprachiasmatic nuclei (SCN) in the anterior hypothalamus. These light-driven signals reset the timing or “phase” of the SCN circadian clocks, which determine the timing of activity and sleep-wake patterns. ^1^ The SCN indirectly synchronizes clocks in peripheral tissues through a combination of neuronal, behavioral, and hormonal cues that are incompletely defined. Disruption of the circadian network due to environmental disturbances, such as that experienced by night shift workers, increases the risk of disease, including malignancy. ^2^ Epidemiological studies observed increased risk of non-Hodgkin’s lymphoma in night shift workers. ^3^ However, rates of B cell and other subtypes of lymphoma were indistinguishable between shift workers and the general population. ^4^ Chronic jetlag (CJL) lighting protocols that mimic the divergent environmental lighting schedule experienced by shift workers have been shown to increase the formation and progression of cancers such as osteosarcoma, hepatocellular carcinomas, breast cancer, and lung adenocarcinoma in mice, ^5^^–^^11^ suggesting that the impact of circadian disruption on tumorigenesis is not constrained by the cell type of origin and independent of oncogenic drivers. Additionally, multiple studies show that people who live further west within a time zone have an increased risk of developing several types of malignancies, including leukemias and lymphomas. ^12^^,^^13^ Nevertheless, the connection between circadian disruption and tumorigenesis remains poorly understood. ^14^

In mammals, the transcription factors brain and muscle ARNT-like protein 1 (BMAL1) and circadian locomotor output cycles kaput (CLOCK) form a heterodimer that activates transcription of genes driven by E-box elements (collectively known as clock-controlled genes, CCGs). CCGs include those that encode periods (PER1-3) and cryptochromes (CRY1-2), which repress BMAL1-CLOCK transactivation activity, resulting in a transcription-translation feedback loop (TTFL) that underpins daily oscillations. ^15^ A secondary TTFL includes nuclear receptor subfamily 1 group D members 1 (NR1D1, a.k.a. REVERBα) and 2 (NR1D2, a.k.a. REVERBβ). BMAL1/CLOCK transactivates *Nr1d1* and *Nr1d2* mRNA expression, and NR1D1 and NR1D2 in turn repress *Bmal1* transcription. ^15^ CCGs comprise thousands of genes, including many involved in proliferation, DNA damage response and repair, and autophagy, which likely contribute to linking dysregulation of the circadian clock to tumorigenesis.

Genetically engineered mouse models (GEMMs) of cancer are valuable research tools utilized to characterize oncogenic or tumor suppressive genes in combination with environmental stressors or novel therapeutic approaches. ^16^ Eμ-MYC mice express human *c-MYC* under the control of the immunoglobulin heavy chain (IgH) enhancer resulting in constitutive activation of *c-MYC* in the B cell lineage. ^17^ Eμ-MYC mice develop spontaneous lymphoma and leukemia in pre-B cells and rapidly succumb to disease. ^18^ *Cry2* deficient Eμ-MYC mice develop an increased number of tumors in the lymph nodes and experience reduced overall survival compared to wildtype littermates. ^19^ This phenomenon is largely attributed to CRY2 post-translational regulation of c-MYC. CRY2, in complex with the SKP-CULLIN-F-box (SCF) E3 ubiquitin ligase containing FBXL3, SCF ^FBXL3^, can bind phosphorylated c-MYC resulting in the polyubiquitination of c-MYC and subsequent proteasomal degradation. ^19^ Here we investigate whether environmental circadian disruption, by means of altered light exposures, influences tumorigenesis in Eμ-MYC mice. We use a disruptive lighting schedule, chronic jetlag (CJL), that we previously demonstrated disrupts rhythmic expression of *Cry2* mRNA in peripheral tissues of C57BL/6J mice. ^11^ Thus, we expected that CJL would enhance tumor development and decrease overall survival in Eμ-MYC mice via loss of CRY2-dependent modulation of c-MYC. To our surprise, and in contrast to all other mouse models of cancer studied to date, CJL impacted neither tumor burden nor survival in Eμ-MYC mice.

## Methods

### Mice

Male Eμ-MYC ^+/-^ mice on a C57BL/6 background were purchased from The Jackson Laboratory at eight weeks of age. Eμ-MYC mice express human *c-MYC* under the control of the immunoglobulin heavy chain (IgH) enhancer resulting in constitutive activation of *c-MYC* in the B cell lineage. ^17^ The Eμ-MYC ^+/-^ mice were housed in the Dorris Neuroscience Center vivarium at Scripps Research and bred with the laboratory colony of C57BL/6 females originally purchased from the Scripps Research breeding colony. All mice used in the research described here were heterozygous for the Eμ-MYC transgene (Eμ-MYC ^+/-^). During breeding for experiments, all mice were included in the study (i.e., no excluded animals). Food and water were provided *ad libitum.* All efforts were made to ameliorate any suffering of the animals involved in this study: mice showing any signs of advanced disease such as grossly visible tumors, rapid breathing, weight loss, etc., were euthanized by CO _2_ inhalation. All animal care and treatments were in accordance with The Scripps Research Institute guidelines for the care and use of animals and were approved by the Institutional Animal Care and Use Committee (IACUC) under protocol number 10-0019.

### Chronic jetlag (CJL) conditions

**Figure 1.**
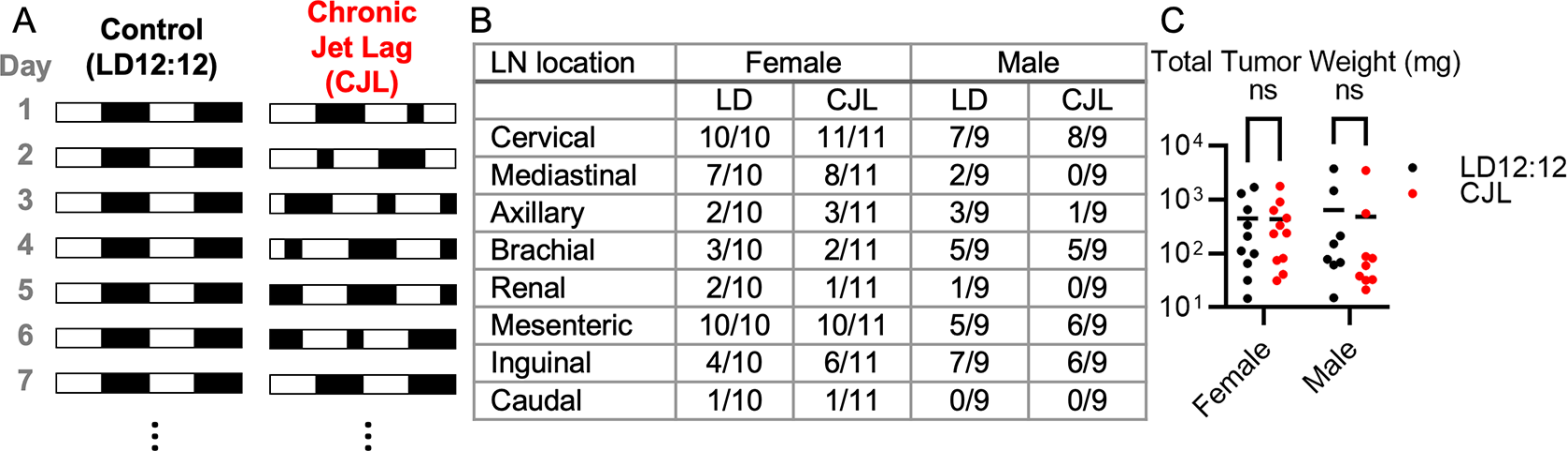
Eight weeks of chronic jetlag does not impact tumor burden in Eμ-MYC mice. (A) Schematic depicting the experimental lighting conditions of the Eμ-MYC mice. The white areas represent periods of lights on, and the black areas represent periods of lights off. Control lighting consists of a rotating 12-hour light phase and 12-hour dark phase (LD12:12) and the chronic jetlag lighting includes an eight-hour phase advance every two to three days (CJL). (B) Fraction of mice with tumors observed at the indicated lymph node (LN) in Eμ-MYC females (left, n=21) and males (right, n=18) housed LD12:12 or CJL lighting conditions for eight weeks. (C) Scatter plot with mean of total tumor weight in female and male Eμ-MYC mice after eight weeks in LD12:12 (black) or CJL (red) lighting conditions. There were no significant (ns, p>0.05) differences between groups by two-way ANOVA.

At four weeks of age, mouse littermates were separated and randomly assigned to light-tight boxes set to either the control (continuous cycles of 12 hours of the light followed by 12 hours of dark, LD12:12) or chronic jetlag (eight-hour light phase advances every two to three days, CJL, ^5^^,^^6^^,^^9^^–^^11^) lighting conditions ( Figure 1A). For the 12-week tumor burden endpoint study, male and female mice were housed in LD12:12 (n=19) or CJL (n=20) for eight weeks before euthanasia at zeitgeber time (ZT, hours after lights on) ZT9 on the day following the first synchronized 24-hour period (i.e., day two of week nine). To minimize potential confounders, dissections of the LD12:12 and CJL were alternated. For the survival study, male and female Eμ-MYC littermates were housed in LD12:12 (n=23) or CJL (n=23) and monitored weekly for signs of advanced disease. For all analyses, the experimental unit is a single animal. Animals found deceased were not assessed for total tumor weight. Sample size was determined by power analysis guided by the expected variability in outcomes using data from Ref. 19. Experimenters were aware of group allocation.

### RNA extraction and quantitative real-time PCR

RNA was extracted from liver and spleen tissue that was flash frozen in liquid nitrogen at the time of sacrifice. One mL of Qiazol reagent (Qiagen cat # 799306) was used to isolate RNA from 50 mg of tissue. Tissue homogenization was achieved by bullet blender tissue homogenizer. 200 μl of chloroform was added to homogenized lysates which were transferred into a phase lock tube (VWR cat # 10847-802). Samples were centrifuged for 15 minutes at 13,000 rmp/4 °C. The aqueous phase was transferred to a new tube and 500 μl of isopropanol was used to precipitate RNA. Samples were centrifuged for 15 minutes at 13,000 rpm/4 °C to pellet RNA. Pellet was washed with 1 mL of 75% ethanol, dried, and diluted in 50 μl nuclease free water. Each sample yielded 2–5 μg/μl of RNA quantified by NanoDrop 2000 spectrophotometer (Thermo scientific cat # ND2000). cDNA was prepared using 1 μg of RNA and 4 μl of QScript cDNA Supermix (VWR cat # 101414-106). Thermocyling conditions were 25 °C for 5 minutes, 42 °C for 30 minutes, and 85 °C for 5 minutes and executed using C1000 Touch Thermal Cycler (Bio-Rad cat # 1851148). cDNA was diluted 1:40 with nuclease free water and 4 μl of diluted cDNA, 5 μl of with iQ SYBR Green Supermix (Bio-Rad cat # 1708885), and 0.5 μl of each forward and reverse primers (10 μM) was used per qPCR reaction. cDNA levels were measured by CFX96 Touch Real Time PCR Detection system (Bio-Rad cat # 1845096). Cycling conditions were, step 1: 95 °C for 3 minutes, step 2: 95 °C for 10 seconds, step 3: 55 °C for 10 seconds, step 4: 72 °C for 30 seconds, step 5: go to step 2 39x, step 6: 95°C for 10 seconds, step 7: melt curve 65–95 °C, increments 0.5 °C for 5 seconds + plate read. Amplification was measured and analyzed by Bio-Rad CFX Manager 3.1. Starting quantity (SQ) as determined by the software was used for statistical analysis. Raw data available. ^20^ The following primers were used to detect *U36b4* (Forward: AGATGCAGCAGATCCGCA *,* Reverse: GTTCTTGCCCATCAGCACC), *Bmal1* (Forward: TCAAGACGACATAGGACACCT, Reverse: GGACATTGGCTAAAACAACAGTG), *P21* (Forward: CCAGGCCAAGATGGTGTCTT, Reverse: TGAGAAAGGATCAGCCAT TGC), *E2f1* (Forward: AGGGAAAGGTGTGAAATCTCC, Reverse: TTGGTGATGACATAGATGCGC).

### Western blot analysis

Crushed liver and spleen tissues were lysed using RIPA buffer supplemented with protease (Thermo Scientific cat # A32953) and phosphatase (Sigma cat # P5266 and cat # P0044) inhibitors. Protein levels were normalized using the Pierce BCA Protein Assay Kit (Thermofisher cat # PI23225). Lysates were separated in an 8% agarose gel by electrophoresis (Bio-Rad cat # 1658001) and transferred using the Trans-blot Turbo transfer system (Bio-Rad cat # 17001915). Membranes were blocked in 5% milk in Tris-buffered saline supplemented with 1% Tween-20 (TBST) for one hour and washed 3X in TBST for 5 minutes before being placed in primary antibodies overnight at 4 °C. Antibodies were diluted 1:1000 for polyclonal antibody raised in rabbit against BMAL1 (Abcam cat # ab93806), polyclonal antibodies raised in guinea pigs against the C-termini of CRY1 (amino acids 583–606) or CRY2 (amino acids 563–592), ^21^ monoclonal antibody raised in rabbit against c-MYC (Abcam cat # ab32072); 1:2,000 for monoclonal antibody raised in mouse against REV-ERBα ^22^; 1:50,000 for monoclonal antibody raised in mouse against β-ACTIN (Sigma cat # A1978) in TBST supplemented with 3% bovine serum albumin (BSA). Membranes were washed 3X in TBST for 5 minutes before incubation in secondary antibody (Goat Anti-Mouse IgG (H + L)-HRP Conjugate (Bio-Rad cat # 1706516), Goat Anti-Rabbit IgG (H + L)-HRP Conjugate (Bio-Rad cat # 1706515), Goat Anti-Guinea Pig IgG-HRP Conjugate (Sigma cat # A7289)) diluted 1:5000 in TBST supplemented with 3% BSA for 1 hour at room temperature. Membranes were washed 3X in TBST for 5 minutes before imaging using SuperSignal West Pico PLUS Chemiluminescent Substrate (Fisher scientific cat # PI34095). Imaging and quantification were performed using the ChemiDoc XRS+ System (Bio-Rad cat # 1708265) and Image Lab software version 6.1.0 build 7. Proteins detected by immunoblotting were normalized to the housekeeping protein β-ACTIN. Brightness and contrast of blots were adjusted using PowerPoint version 2022. Any and all adjustments that were made were applied to the entire image. Raw data are available. ^20^

### Statistical analysis

Statistical analyses were performed using GraphPad Prism 8 software. The statistics for this research could be reproduced using open-source graphical program for statistical analysis JASP. Significance for total tumor weight from the tumor burden and survival cohorts were determined by two-way ANOVA; qPCR and Western blots were determined by t-test; Kaplan-meier survival curves were determined by Log-rank (Mantel-Cox) test. Significance threshold was set at 0.05 acceptable false positive (p<0.05).

## Results

### Chronic jetlag does not impact tumor burden in Eμ-MYC mice

Exposure to circadian disruption through altered lighting enhances tumor growth and reduces overall survival in c57BL6/J wildtype mice (due to increased spontaneous development of hepatocellular carcinoma) and in genetically engineered mouse models (GEMMs) of cancer. ^5^^–^^7^^,^^9^^–^^11^^,^^23^ We chose to use a chronic jetlag (CJL) protocol that has been used in several of these studies. ^5^^,^^6^^,^^9^^–^^11^ Throughout this study, CJL denotes a lighting schedule in which the lights are turned on eight hours earlier (i.e., the light phase is advanced by eight hours) every two to three days ( Figure 1A) to mimic circadian disruption experienced by rotating shift workers.

Eμ-MYC mice were housed in either CJL or control (12 hours of light followed by 12 hours of dark, indefinitely; LD12:12) lighting conditions at four weeks of age ( Figure 1A). Mice were maintained in CJL or LD12:12 lighting conditions for eight weeks before euthanasia at zeitgeber time (ZT, hours after lights on) ZT9 on the day following the first synchronized 24-hour period (i.e., day 2 of week 9). Four mice were excluded from the study because they died before the designated endpoint. CJL affected neither the tumor spectrum ( Figure 1B) nor overall tumor burden as revealed by the combined weight of all tumors dissected from each animal ( Figure 1C) in male or female Eμ-MYC mice.

### Exposure to long-term CJL impacts neither tumor burden nor survival of Eμ-MYC mice

**Figure 2.**
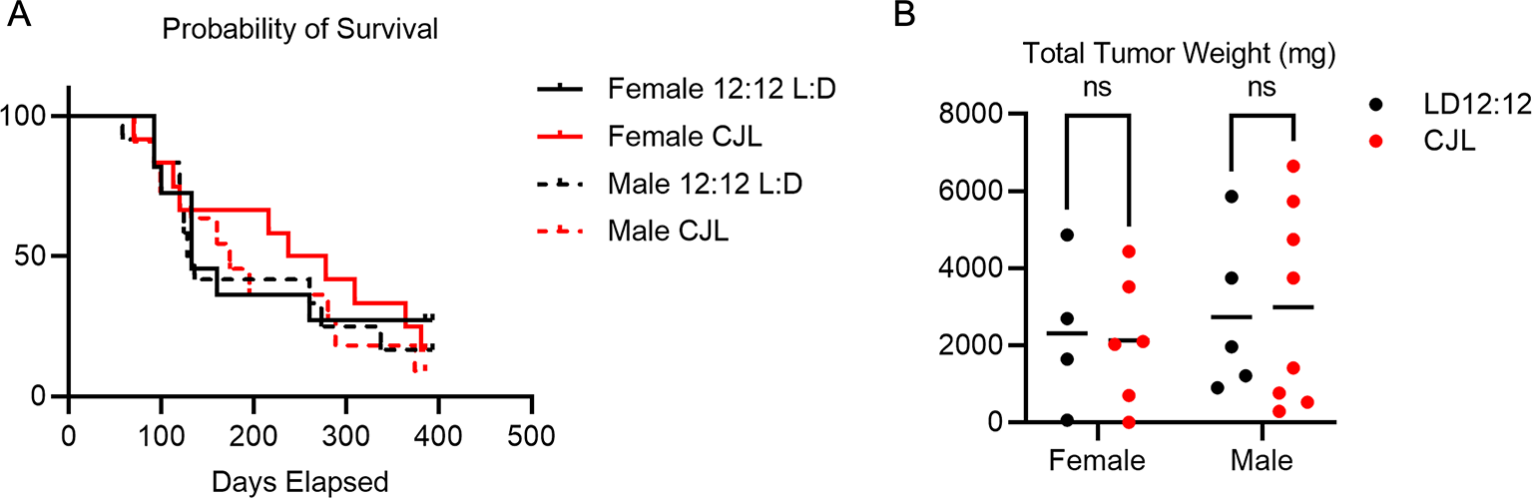
Long term chronic jetlag does not impact tumor burden or survival of Eμ-MYC mice. (A) Kaplan-Meier survival curves for female Eμ-MYC mice housed in LD12:12 (solid black, n=11) or in CJL (solid red, n=12) and male Eμ-MYC mice housed in LD12:12 (dashed black, n=12) or in CJL (dashed red, n=11). (B) Scatter plot with mean of total tumor weight at the time of euthanasia of female Eμ-MYC mice housed in LD12:12 (black, n=4) or in CJL (red, n=5) and male Eμ-MYC mice housed in LD12:12 (black, n=5) or in CJL (red, n=8). There were no significant (ns, p>0.05) differences between groups by logrank test (A) or by two-way ANOVA (B).

At four weeks of age, female and male Eμ-MYC mice were placed in either LD12:12 or CJL lighting conditions. Mice were housed in these conditions until they exhibited signs of advanced disease (e.g., grossly visible tumors, rapid breathing), at which point mice were euthanized and total tumor weight was recorded. There was no significant difference in the overall survival ( Figure 2A) or the terminal tumor weight ( Figure 2B) of male or female Eμ-MYC mice exposed to CJL compared to those housed in control lighting conditions.

### CJL disrupts circadian rhythms in peripheral tissues

**Figure 3.**
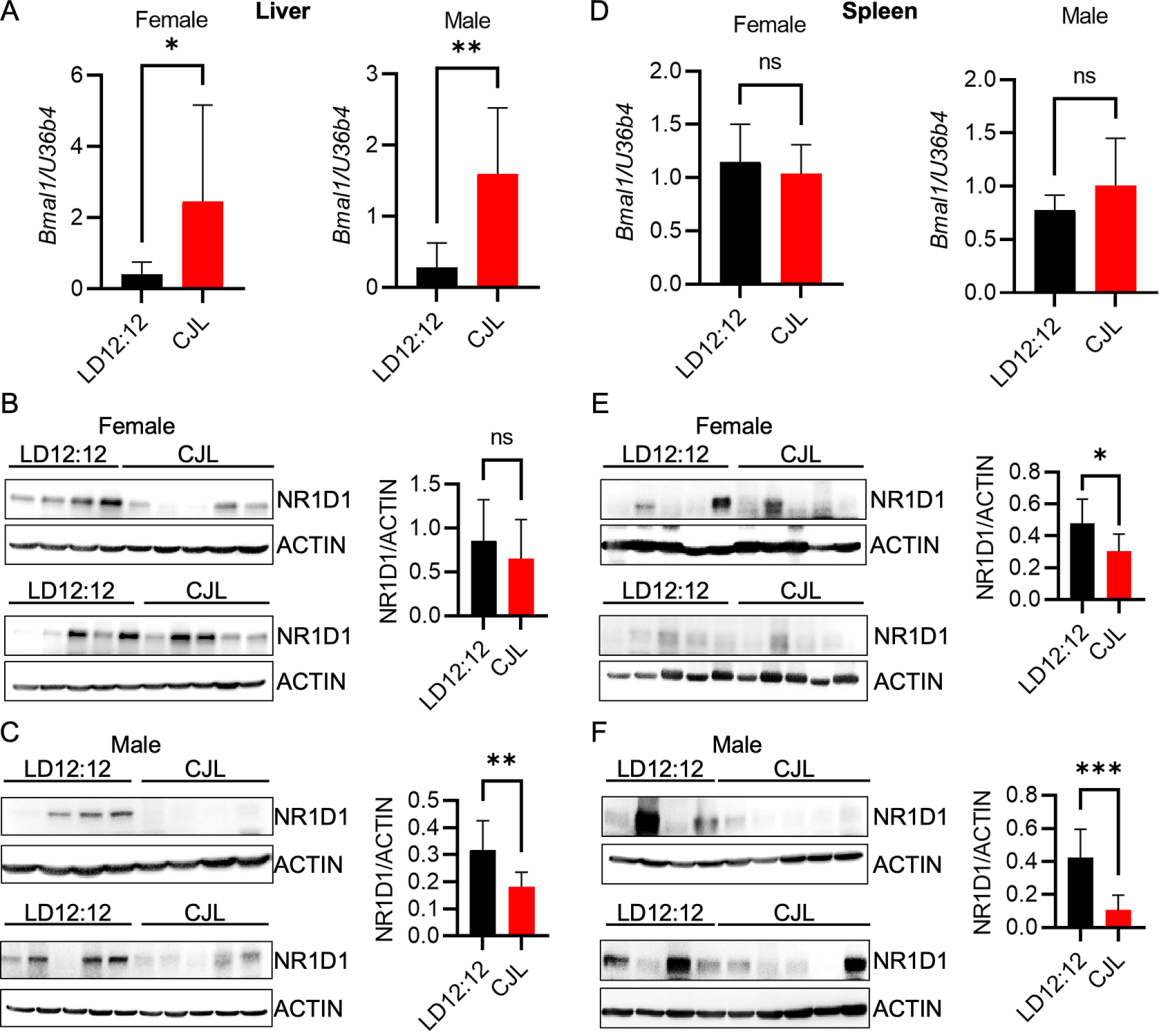
Chronic jetlag alters the clock in the peripheral tissues of Eμ-MYC mice. (A–F) Detection of the indicated transcripts by qPCR (A,D) and proteins by immunoblotting (B,C,E,F) in liver (A–C) or spleen (D–F) tissues collected at ZT9 from Eμ-MYC mice housed in LD12:12 or CJL lighting conditions for eight weeks. Error bars indicate standard deviation. *p<0.05, **p<0.01, ***p<0.001, or not significant (ns, p>0.05) by *t-*test.

We previously demonstrated that CJL disrupts locomotor activity rhythms in c57BL/6J mice and alters rhythmic gene expression patterns in peripheral tissues of both healthy and tumor-bearing mice. ^11^ To examine the impact of CJL on gene expression in the context of MYC-driven lymphoma, we euthanized Eμ-MYC mice at ZT9, when *Bmal1* mRNA is typically low and REV-ERBα protein is typically high in peripheral organs. Livers and spleens were flash frozen at the time of dissection to evaluate molecular circadian rhythms. Consistent with findings in healthy mice, ^11^ *Bmal1* mRNA was significantly increased at ZT9 in samples prepared from livers of male and female Eμ-MYC mice that had been exposed to CJL compared to littermates housed in control LD12:12 lighting conditions ( Figure 3A). Conversely, NR1D1 protein tended to be lower in liver tissue collected at ZT9 from mice housed in CJL compared to the control group ( Figure 3B, C).

In Eμ-MYC mice, human *c-MYC* is expressed at a high level in B cells via transgenic expression under the control of the IgH enhancer resulting in generation of MYC-driven lymphomas. ^17^ The spleen is the site of B cell maturation and we have previously shown that deletion of the clock component *Cry2* leads to enhanced expression of MYC in the spleen of Eμ-MYC mice. ^19^ Thus, we assessed the impact of circadian disruption in the spleen tissues of the Eμ-MYC mice. There was no significant difference in *Bmal1* transcript levels in the spleen tissue from mice exposed to CJL compared to those housed in control lighting conditions ( Figure 3D), in contrast to the significant elevation in liver of the same animals ( Figure 3A). NR1D1 protein was significantly lower in spleens collected from mice that had been exposed to CJL, regardless of sex ( Figure 3E, F).

### CJL does not alter c-MYC in liver or spleen

**Figure 4.**
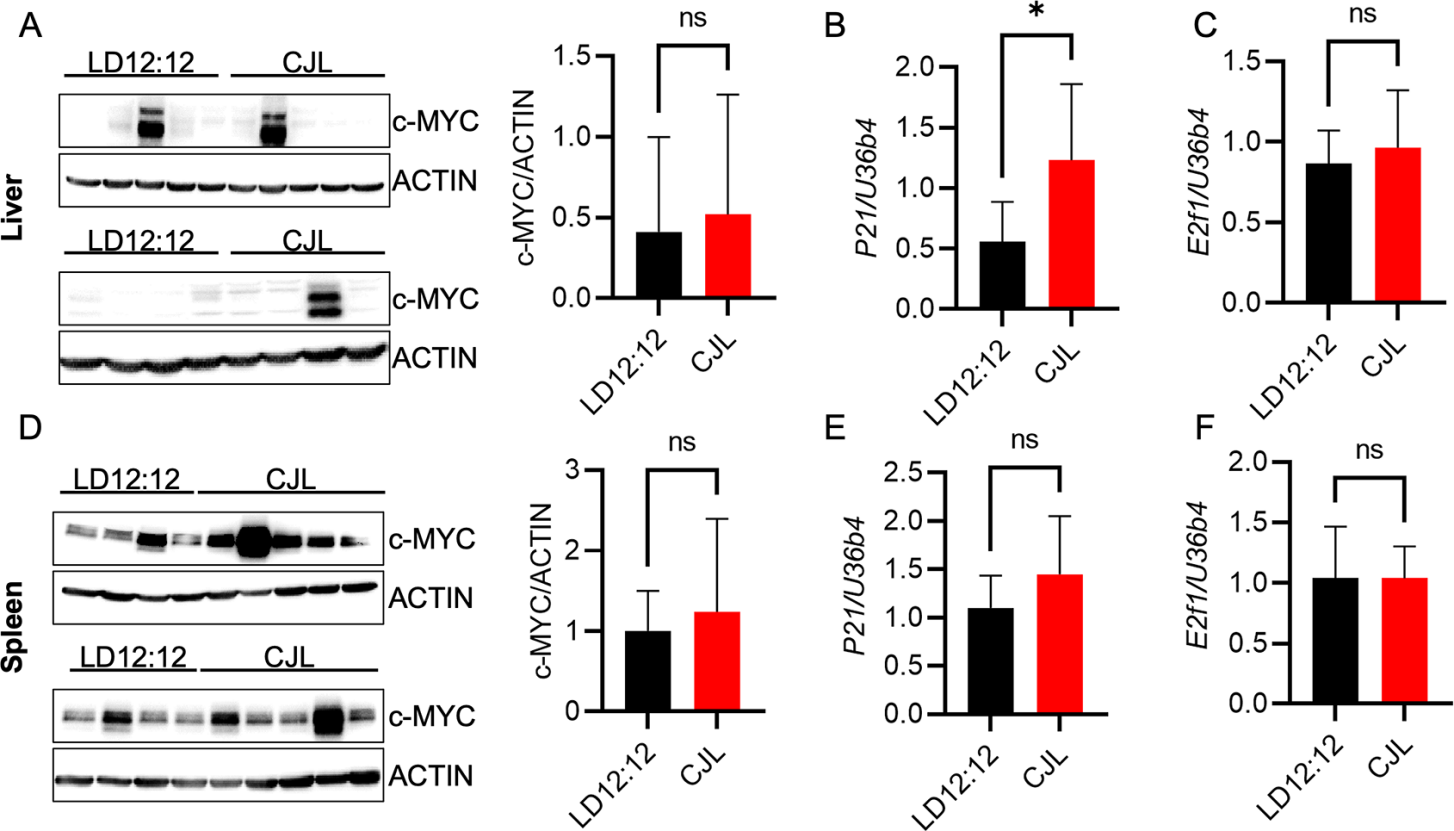
Chronic jetlag has negligible impact on c-MYC in peripheral tissues of Eμ-MYC mice. (A–F) Detection of the indicated proteins by immunoblotting (A, D) and transcripts by qPCR (B,C,E,F) in liver (A–C) or spleen (D–F) tissues collected at ZT9 from male Eμ-MYC mice housed in LD12:12 or CJL lighting conditions for eight weeks. Error bars indicate standard deviation. *p<0.05 or not significant (ns, p>0.05) by *t-*test.

We assessed the impact of CJL on c-MYC protein levels and expression of c-MYC transcriptional targets, *P21* and *E2f1*, in the peripheral tissues of Eμ-MYC mice. c-MYC protein levels were highly variable and were not significantly different in liver tissues of mice housed in CJL compared to those housed in LD12:12 ( Figure 4A). There was a significant increase in expression of *P21* in liver tissue from mice housed in CJL relative to those housed in LD12:12 ( Figure 4B). This is consistent with previous reports of circadian regulation of *P21* in murine hepatocytes. ^24^ There was no difference in *E2f1* expression in livers collected from mice housed in control or CJL conditions ( Figure 4C). Similarly, neither c-MYC protein ( Figure 4D) nor expression of the MYC target genes *P21* ( Figure 4E) and *E2f1* ( Figure 4F) was affected by CJL in the spleen tissue of male Eμ-MYC mice. Taken together, these findings show that CJL disrupts molecular circadian rhythms in liver and spleen tissues of Eμ-MYC mice.

## Discussion

Epidemiological research supports the idea that disruption of circadian rhythms, such as that experienced by shift workers, increases the incidence of several types of cancer. ^5^^–^^10^ The evidence for such a connection is strongest for breast cancer, due at least in part to the volume of research performed in that area. A recent study found that the incidence of chronic lymphocytic leukemia is increased among those who have ever done night shift work, but rates of B cell lymphoma and other subtypes of lymphoma were indistinguishable between shift workers and the general population. ^4^ An earlier study reported increased risk of non-Hodgkin’s lymphoma in shift workers. ^3^ One limitation of epidemiological studies is the large variability in human lifestyles and genetics; thus, mouse models of cancer provide an alternative approach with reduced heterogeneity. Almost 20 years ago, human tumor xenografts were found to grow faster when transplanted into mice in which the suprachiasmatic nuclei were destroyed. ^25^ To better mimic chronic disruption of a functional circadian timing system experienced by shift workers, circadian biologists have widely adopted the use of altered lighting schedules, broadly referred to as chronic jetlag (CJL). ^5^^,^^7^^,^^8^^,^^10^^,^^11^^,^^26^^–^^30^ Several studies have demonstrated that various types of CJL increase the tumor burden in mouse models of cancer. ^7^^,^^8^^,^^10^^,^^11^^,^^26^^,^^27^

Further supporting the notion that circadian disruption broadly enhances tumorigenesis, genetic disruption of various circadian clock components enhances tumor burden in mouse models of cancer. ^7^^,^^9^^,^^23^^,^^31^^–^^36^ However, a few contrary examples in which inactivation of *Bmal1* or of both *Cry1* and *Cry2* reduced tumor formation ^35^^,^^37^ illustrate the error in considering either circadian disruption or cancer as monolith. ^38^

To understand the impact of environmental circadian disruption on tumorigenesis, it is essential to recognize how disruption of regular light exposures impacts malignancy in a variety of contexts. This study specifically assessed the impact of CJL on the Eμ-MYC mouse model of lymphoma, based on previous reports demonstrating that CRY2 facilitates the turnover of c-MYC and that genetic depletion of *Cry2* in Eμ-MYC mice enhances tumorigenesis. ^19^ Here, we explored the possibility that environmental circadian disruption would likewise enhance c-MYC accumulation and tumor burden in Eμ-MYC mice.

Our established protocol of CJL disrupts circadian expression of *Cry2* mRNA in peripheral tissues of wildtype C57BL/6J mice. ^11^ Therefore, we anticipated that CJL would influence c-MYC in Eμ-MYC mice by disrupting CRY2-mediated turnover of c-MYC and would thus alter the progression of MYC-driven lymphoma. Unexpectedly, we measured no difference in the number of lymph node tumors or overall survival of Eμ-MYC mice housed in CJL conditions compared to their littermates maintained in standard LD12:12 light-dark cycles.

While we measured disruption of molecular circadian rhythms in liver and spleen tissue collected from Eμ-MYC mice that were housed in CJL, the impact of CJL seems to be less severe in spleens. Interestingly, we also observed a less severe impact of CJL on circadian gene expression in spleens of healthy mice than in their livers. ^11^ The influence of circadian disruption in spleens of Eμ-MYC mice may be further subdued by the aggressiveness ^18^ or the intrinsic heterogeneity ^39^^,^^40^ of Eμ-MYC lymphoma. Eμ-MYC mice can develop tumors with biological similarities to Burkitt lymphoma or diffuse large B cell lymphoma that exhibit divergent activation of c-MYC. ^40^ This is consistent with our observations of variable levels of c-MYC protein in the liver ( Figure 4A) and spleen ( Figure 4D) of the Eμ-MYC mice independent of lighting condition. The heterogeneity in c-MYC levels was an unexpected confounding factor in this study. Nevertheless, given that spleen is the site for B cell maturation, the apparently lower sensitivity of spleen to circadian disruption caused by CJL may contribute to the lack of impact on B cell lymphoma observed in this study.

## Conclusions

Our findings suggest that environmental circadian disruption similar to that experienced by shift workers does not influence MYC-driven lymphomagenesis in a c57BL/6J mouse model. Given the strong evidence that altered light exposures can impact lymphoid cancers in people, additional investigation is needed to identify the mechanistic underpinning for this phenomenon that is not present in the mouse model studied here.

## Data Availability

Figshare: Underlying data for ‘Circadian disruption does not alter tumorigenesis in a mouse model of lymphoma’, https://doi.org/10.6084/m9.figshare.20492892.v3. ^20^ This project contains the following underlying data:
• Data file 1: EuMYC_F_TumorBurden_BMAL1_Liver.xlsx • Data file 2: EuMYC_F_TumorBurden_Bmal1_Spleen.xlsx • Data file 3: EuMYC_F_TumorBurden_U36b4_Liver.xlsx • Data file 4: EuMYC_F_TumorBurden_U36b4_Spleen.xlsx • Data file 5: EuMYC_M_Tumorbuden_Liver_Bmal1.xlsx • Data file 6: EuMYC_M_Tumorbuden_Liver_E2f1.xlsx • Data file 7: EuMYC_M_Tumorbuden_Liver_P21.xlsx • Data file 8: EuMYC_M_Tumorbuden_Liver_U36b4.xlsx • Data file 9: EuMYC_M_Tumorbuden_Spleen_Bmal1.xlsx • Data file 10: EuMYC_M_Tumorbuden_Spleen_E2F1.xlsx • Data file 11: EuMYC_M_Tumorbuden_Spleen_p21.xlsx • Data file 12: EuMYC_M_Tumorbuden_Spleen_U36b4.xlsx • Data file 13: Mouse_Information.xlsx • Supplementary file 1: Eu-MYC_F_TB_Liver_Actin_1_merge.tif • Supplementary file 2: Eu-MYC_F_TB_Liver_Actin_2_merge.tif • Supplementary file 3: Eu-MYC_F_TB_Liver_NR1D1_1_merge.tif • Supplementary file 4: Eu-MYC_F_TB_Liver_NR1D1_merge.tif • Supplementary file 5: Eu-MYC_M_TB_Liver_Actin_1_merge.tif • Supplementary file 6: Eu-MYC_M_TB_Liver_Actin_2_merge.tif • Supplementary file 7: Eu-MYC_M_TB_Liver_c-MYC_1_merge.tif • Supplementary file 8: Eu-MYC_M_TB_Liver_c-MYC_2_merge.tif • Supplementary file 9: Eu-MYC_M_TB_Liver_NR1D1_1_merge.tif • Supplementary file 10: Eu-MYC_M_TB_Liver_NR1D1_2_merge.tif • Supplementary file 11: EuMYC_M_TB_Spleen_gel1_ACTIN_merge.tif • Supplementary file 12: EuMYC_M_TB_Spleen_gel1_MYC_2_merge.tif • Supplementary file 13: EuMYC_M_TB_Spleen_gel1_NR1D1_merge.tif • Supplementary file 14: EuMYC_M_TB_Spleen_gel2_ACTIN_merge.tif • Supplementary file 15: EuMYC_M_TB_Spleen_gel2_MYC_2_merge.tif • Supplementary file 16: EuMYC_M_TB_Spleen_gel2_NR1D1_merge.tif • Supplementary file 17: EuMYC_TB_Spleen_F_ACTIN_gel6_merge[32504].tif • Supplementary file 18: EuMYC_TB_Spleen_F_NR1D1_gel6_merge[32506].tif • Supplementary file 19: TumorBurden_F_gel4_actin_merge[32512].tif Repository: ARRIVE checklist for ‘Circadian disruption does not alter tumorigenesis in a mouse model of lymphoma’, https://doi.org/10.6084/m9.figshare.20492892.v3. ^20^ Data are available under the terms of the Creative Commons Attribution 4.0 International license (CC-BY 4.0).
