# Supplementary material for: Circadian disruption does not alter tumorigenesis in a mouse model of lymphoma
Source: F1000Res. 2023 Sep 28;12:49. Originally published 2023 Jan 12. [Version 2] doi: 10.12688/f1000research.125272.2 (PMC10558980; doi:10.12688/f1000research.125272.2)
Supplement: Peer review history

# Reviewer report: Reviewer response for version 2

**DOI:** 10.5256/f1000research.156285.r210637

**Published:** 2023-10-06

**Authors:** Eric Zhang

**Affiliations:** 1 National Institute of Biological Sciences (NIBS), Beijing, China

My concerns from last review have been fully addressed.

Is the work clearly and accurately presented and does it cite the current literature?

Yes

If applicable, is the statistical analysis and its interpretation appropriate?

Partly

Are all the source data underlying the results available to ensure full reproducibility?

Yes

Is the study design appropriate and is the work technically sound?

Yes

Are the conclusions drawn adequately supported by the results?

Partly

Are sufficient details of methods and analysis provided to allow replication by others?

Yes

Reviewer Expertise:

circadian rhythms, physiology, neuroscience

I confirm that I have read this submission and believe that I have an appropriate level of expertise to confirm that it is of an acceptable scientific standard.

# Reviewer report: Reviewer response for version 2

**DOI:** 10.5256/f1000research.156285.r210636

**Published:** 2023-10-06

**Authors:** Chi Van Dang

**Affiliations:** 1 Ludwig Institute for Cancer Research, New York, NY, USA

The authors have revised the manuscript and include an expanded discussion.

Is the work clearly and accurately presented and does it cite the current literature?

Partly

If applicable, is the statistical analysis and its interpretation appropriate?

Partly

Are all the source data underlying the results available to ensure full reproducibility?

No

Is the study design appropriate and is the work technically sound?

Yes

Are the conclusions drawn adequately supported by the results?

Partly

Are sufficient details of methods and analysis provided to allow replication by others?

No

Reviewer Expertise:

molecular and cell biology of cancer

I confirm that I have read this submission and believe that I have an appropriate level of expertise to confirm that it is of an acceptable scientific standard.

# Reviewer report: Reviewer response for version 1

**DOI:** 10.5256/f1000research.137559.r160078

**Published:** 2023-01-30

**Authors:** Eric Zhang

**Affiliations:** 1 National Institute of Biological Sciences (NIBS), Beijing, China

**General comments**

 This study described a phenomenon that 8-week chronic jet lag, which was reported to disrupt circadian rhythm in C57BL/6J mice, did not exacerbate lymphomas in Eμ-MYC mice. The results and conclusions are striking, for it is widely accepted that disruption of circadian rhythm could promote the progression of cancers, and such influences have been confirmed in animal models and number of clinical studies. As the authors indicated in the Conclusions part, it would be important and interesting if the mechanism under this phenomenon is investigated. As for the current version, more experimental data is needed to strengthen each part of the conclusions.

**Major points:**

 1. Survival curve and total tumor weight are the only two indicators to support that long-term CJL does not impact the progress of lymphomas in Eμ-MYC mice. To address tumor burden, it will be better to have photos of tumor samples in addition to the statistical data. And other pathological analysis of lymphoma and the overall health of mice are needed to give a more detailed description on the cancer progression.

 2. Although there are evidences that such chronic jet lag protocol could disrupt the rhythm of C57BL/6J mice, how it works on Eμ-MYC mice still needs to be addressed in this paper, and it is also necessary to show the original rhythmicity of Eμ-MYC mice under LD/DD condition. Although the authors explained why they sampled at ZT9, a time course sampling (or at least anti-phase sampling) could better describe the influence of CJL on the peripheral tissues in Eμ-MYC mice.

 3. At first, based on the phenotypes in *Cry2* KO mice, the authors hypothesized that CJL may also promote the progression of cancer in Eμ-MYC mice due to the disruption of circadian clock, but surprisingly found the opposite results. However, the disruption of circadian clock induced by CJL is different from *Cry2* KO. For in the reference 11, the core clock genes such as *Bmal1*, *Cry1*, *Cry2*, and *Per2*, showed a different phase after CJL with remained, though dampened rhythmicity, while it is not like that in *Cry2 *KO mice. It is likely that the altered c-MYC is downstream of decreased CRY2 in *Cry2 *KO mice, and in Eμ-MYC mice, the expression level of c-MYC itself is variable, so *Cry2* may not play a role in the case of Eμ-MYC mice.

 Therefore, this reviewer suggests the authors to make some rearrangement on this article, especially the Background and Discussion parts, in order to focus on the phenomenon of this article.

**Minor points:**

 1. Fig.1C and Fig.2B described the same factor, so it will be better to use the same range of y axis.

 2. The data quality of Fig.3E could be improved, for Actin is not consistent now.

 3. It will be better for the authors to make some preliminary hypothesis of the mechanism according to previous and the current studies in the Discussion part.

Is the work clearly and accurately presented and does it cite the current literature?

Yes

If applicable, is the statistical analysis and its interpretation appropriate?

Partly

Are all the source data underlying the results available to ensure full reproducibility?

Yes

Is the study design appropriate and is the work technically sound?

Yes

Are the conclusions drawn adequately supported by the results?

Partly

Are sufficient details of methods and analysis provided to allow replication by others?

Yes

Reviewer Expertise:

circadian rhythms, physiology, neuroscience

I confirm that I have read this submission and believe that I have an appropriate level of expertise to confirm that it is of an acceptable scientific standard, however I have significant reservations, as outlined above.

# Reviewer report: Reviewer response for version 1

**DOI:** 10.5256/f1000research.137559.r160077

**Published:** 2023-01-25

**Authors:** Chi Van Dang

**Affiliations:** 1 Ludwig Institute for Cancer Research, New York, NY, USA

The manuscript by Mello et al. reports experiments that document the lack of an effect of circadian disruption by chronic jet-lag lighting protocol (CJL) on the development and progression of lymphoma in the Eu-Myc transgenic model. The authors previously reported that homozygous loss of Cry2, which mediates Myc protein degradation, increased Eu-Myc tumorigenesis with shorten survival. Hence, with CJL, which decreases the expression of Cry2, they surmised that CJL would enhance tumorigenesis and phenocopy genetic homozygous loss of Cry2. The intention of the study is laudable, however, the assumption that hypomorphic Cry2 (ie, via CJL) would mimic Cry2 null state seems non-compelling. Specifically, in the authors’ 2016 Molecular Cell paper, crossings were performed of EuMyc+/-;Cry2+/- males to Cry2+/- females to generate EuMyc+/-;Cry2+/+ and EuMyc+/-;Cry2-/- mice for analysis. The authors did not report the fate of the EuMyc+/-;Cry2+/- hemizygous Cry2 males compared to EuMyc+/-;Cry2-/- homozygous Cry2 loss males. It would be instructive, if the authors have the old data to report the survival of the Cry+/- hemizygous Cry2 EuMyc mice. This is particularly important, because the dosage effect of Cry2 could be the major driver of the EuMyc model unrelated to circadian disruption (eg. CJL) per se.

 Although the findings of the manuscript by Mello et al. are negative, they are important to document in the literature. However, the distinction between hemizygous and homozygous loss of Cry2 should be explicitly stated so that the study is more instructive for readers.

 Minor:

1. Figure 2. Please comment on whether there are any survival differences between female CJL and female 12:12 L:D mice if the experiment was terminated at 200 days.
2. Figures 3 and 4. Please define the immunoblot lanes; presumably these represent individual animals. If so, please add to the figure legend. The authors may want to comment on the variability of Myc levels in the individual spleens, which should have lymphoma, and whether the extent of variability may mask any potential effect of CJL. For the associated bar graphs, please indicate the sample size.
3. References 9 and 27 are duplicates.

Is the work clearly and accurately presented and does it cite the current literature?

Partly

If applicable, is the statistical analysis and its interpretation appropriate?

Partly

Are all the source data underlying the results available to ensure full reproducibility?

No

Is the study design appropriate and is the work technically sound?

Yes

Are the conclusions drawn adequately supported by the results?

Partly

Are sufficient details of methods and analysis provided to allow replication by others?

No

Reviewer Expertise:

molecular and cell biology of cancer

I confirm that I have read this submission and believe that I have an appropriate level of expertise to state that I do not consider it to be of an acceptable scientific standard, for reasons outlined above.
